# An fMRI Investigation into the Effects of Ketogenic Medium-Chain Triglycerides on Cognitive Function in Elderly Adults: A Pilot Study

**DOI:** 10.3390/nu13072134

**Published:** 2021-06-22

**Authors:** Yukihito Yomogida, Junko Matsuo, Ikki Ishida, Miho Ota, Kentaro Nakamura, Kinya Ashida, Hiroshi Kunugi

**Affiliations:** 1 National Center of Neurology and Psychiatry, Department of Mental Disorder Research, National Institute of Neuroscience, Tokyo 187-8502, Japan; matsujun@ncnp.go.jp (J.M.); iishida@ncnp.go.jp (I.I.); ota@ncnp.go.jp (M.O.); hkunugi@med.teikyo-u.ac.jp (H.K.); 2 Food Microbiology & Function Research Laboratories, R & D Division, Meiji Co., Ltd., Tokyo 192-0919, Japan; kentarou.nakamura@meiji.com (K.N.); kinya.ashida@meiji.com (K.A.); 3 Department of Psychiatry, Teikyo University School of Medicine, Tokyo 173-8605, Japan

**Keywords:** β-hydroxybutyrate, cognition, elderly, ketogenic meal, medium-chain triglycerides, fMRI, VBM

## Abstract

Evidence suggests that oral intake of medium-chain triglycerides (MCTs), which promote the production of ketone bodies, may improve cognitive functions in elderly people; however, the underlying brain mechanisms remain elusive. We tested the hypothesis that cognitive improvement accompanies physiological changes in the brain and reflects the use of ketone bodies as an extra energy source. To this end, by using functional magnetic resonance imaging, cerebral blood oxygenation level-dependent (BOLD) signals were measured while 20 healthy elderly subjects (14 females and 6 males; mean age: 65.7 ± 3.9 years) were engaged in executive function tasks (N-back and Go-Nogo) after ingesting a single MCT meal (Ketonformula^®^) or placebo meal in a randomized, double-blind placebo-controlled design (UMIN000031539). Morphological characteristics of the brain were also examined in relation to the effects of an MCT meal. The MCT meal improved N-back task performance, and this was prominent in subjects who had reduced grey matter volume in the dorsolateral prefrontal cortex (DLPFC), a region known to promote executive functions. When the participants were dichotomized into high/low level groups of global cognitive function at baseline, the high group showed improved N-back task performance, while the low group showed improved Go-Nogo task performance. This was accompanied by decreased BOLD signals in the DLPFC, indicative of the consumption of ketone bodies as an extra energy source.

## 1. Introduction

With today’s increasing life expectancy, it is increasingly important for the elderly to preserve and improve their cognitive functions, which are vital for maintaining independent living and quality of life [1,2]. Recently, it has been suggested that elderly people can improve their cognitive functions by ingesting specific meals that elevate plasma ketone body levels, and in this context, the oral intake of medium-chain triglycerides (MCTs) has been garnering considerable attention [3,4,5]; however, its physiological mechanisms in the brain remain elusive.

Usually, the brain utilizes glucose as its primary energy source. However, when the glucose supply is limited, such as during prolonged fasting, ketone bodies made from fatty acids in the liver become an extra energy source [6]. There are three types of ketone bodies: acetone, β-hydroxybutyrate, and acetoacetate. Of these, the latter two are utilized in the brain [7]. Ketone bodies are recognized as having neuroprotective effects, and promoting ketone body production through a low-carbohydrate diet has been used clinically for intractable epilepsy [8]. Recently, increasing evidence has suggested that ketone bodies are also effective for the treatment of other neurological disorders. Longitudinal studies have shown that elevating the plasma ketone body level improved cognitive dysfunction in people suffering from Alzheimer’s disease [9,10,11] and mild cognitive impairment [12,13]. In these cases, oral intake of MCTs has often been selected as the means to elevate plasma ketone body levels [4]. MCTs, the main constituent of coconut and palm kernel oil, are triglycerides comprising fatty acid chains of between 5 and 12 carbons. When consumed, MCTs are rapidly absorbed and quickly metabolized into medium-chain fatty acids, which in turn promote the generation of ketone bodies in the liver [14,15,16]. Since this process occurs regardless of other macronutrients consumed, consuming MCTs can increase ketone bodies without restricting carbohydrate and protein levels in foods. This makes compliance with this diet easy for a wide range of people. In the case of mild cognitive impairment, a crossover study showed that even a single administration of an MCT meal was associated with better cognitive performance [5]. A similar result was reported in a recent study that targeted healthy elderly individuals without dementia [3]. These studies indicate the possibility that the beneficial effect of an MCT meal on cognitive function emerges very rapidly, at least in elderly people without severe cognitive impairment. Regarding the mechanisms of such a rapid effect on cognitive function, authors of studies have raised the possibility that changes in cognitive performance occur because MCTs effectively increase ketone bodies in the brain, and then, these ketone bodies are used as an extra energy source that supplements energy production by glucose [3,5]. This idea is consistent with the fact that impaired glucose utilization and spared ketone body utilization have been widely observed in the brains of elderly individuals, especially those with cognitive impairment [17,18,19,20,21]. To our knowledge, however, no study has assessed the physiological changes in the brain at exactly the time when elderly subjects are engaged in cognitive activities on which the performance changes with MCT intake. Hence, the detailed physiological mechanisms in the brain that associate MCT intake with better cognitive performances remain elusive.

In the present study, we aimed to assess the hypothesis that when elderly people show better performance in some cognitive functions after taking MCTs, the brain regions responsible for those functions show physiological changes reflecting the use of ketone bodies as an extra energy source. This hypothesis was tested by conducting a double-blind placebo-controlled study in which cerebral blood oxygenation level-dependent (BOLD) signals were measured when elderly subjects were engaged in cognitive tasks after ingesting a meal containing MCTs (MCT meal) or a meal containing placebo, using functional magnetic resonance imaging (fMRI). Since fMRI BOLD signals reflect the degree of increase in regional cerebral blood flow (CBF) triggered by glucose consumption in neural activities [22], a reduced BOLD signal was expected when ketone bodies were consumed as an extra energy source. To measure elderly people’s cognitive performance and corresponding BOLD signals, we employed tasks that target executive functions, since impairment of these functions characterizes normal and pathological age-related cognitive decline [23,24]. Regarding the brain regions responsible for executive functions, previous functional and morphological neuroimaging studies have suggested that the dorsolateral prefrontal cortex (DLPFC) plays a pivotal role [25,26], and this region’s activity is known to show age-related changes [27]. Hence, we focused our analysis on this region. Furthermore, we not only analysed data from all participants together but also conducted stratified analyses in which participants were grouped based on their level of global cognitive function, since this factor is known to affect brain activities in elderly people [28,29]. In addition, we examined the possible association between the effects of the MCT meal and morphological characteristics of the brain by using voxel-based morphometry (VBM) analysis on regional grey matter volume, which has been widely used to explore the neural basis of diverse human characteristics, including responses to pharmacological treatments [20,30,31].

## 2. Materials and Methods

### 2.1. Participants

The participants were 20 elderly volunteers (14 females and 6 males; age: 65.7 ± 3.9 years, range: 60–74 years; Table 1). In determining the optimal sample size, we could not conduct a power analysis based on data from previous studies that employed similar crossover designs to ours [3,5]. This was because the face-to-face cognitive tasks used in those studies were not readily conducted with MRI, and we employed different fMRI-compatible executive function tasks (as detailed in Section 2.3). Hence, in this pilot study, we chose to follow these previous studies and recruited 20 participants (as both previous studies employed 20 participants). All participants had to be free of any psychiatric disorder as assessed with the Japanese version of the Mini-International Neuropsychiatric Interview (MINI) [32,33]. Moreover, all participants were confirmed to be right-handed as assessed with the Edinburgh Handedness Inventory [34] to avoid including participants who potentially have a reversed-laterality brain activation pattern, which could confound the fMRI analysis [35]. The participants also completed the Japanese version of the Mini-Mental State Examination (MMSE) [36,37] to assess global cognitive function level. Participants who scored less than 26 on the MMSE were not enrolled in the study (no subject was excluded on this basis). Other exclusion criteria included (i) MRI contraindications; (ii) history of central nervous system disease or severe head injury; (iii) allergy to milk, soy, and pork that were contained in the test MCT meal; and (iv) lactose intolerance. Written informed consent was obtained from all participants prior to participation. This study was approved by the ethics committee of the National Center of Neurology and Psychiatry, Japan, and by the Meiji institutional review board, Japan, in accordance with the ethical standards laid down in the 1964 Declaration of Helsinki and its later amendments.

### 2.2. Procedure

This study was conducted with a double-blind placebo-controlled design with two study visits. During each visit, participants received one of two isocaloric (371 kcal) meals in a randomized order: a serving of the MCT meal containing emulsified MCTs or a placebo meal containing emulsified long-chain triglycerides as a substitute for MCTs. Except for this difference, the compositions of the MCT meal and placebo meal were exactly the same (protein, carbohydrate, lipid, etc.). Meiji817-B (Ketonformula^®^; Meiji Co., Ltd., Tokyo, Japan), which is a formula approved for ketogenic diet therapies, was used as the MCT meal (see Table 2 for its composition). Test meals were served to participants by an experimenter (YY) who was blind to the meal condition. This blindness was achieved based on the fact that the meals were indistinguishable from each other by appearance and were delivered to the experimenter using the same package design. For each visit, the experimenter received a package that randomly contained an MCT or placebo meal from Meiji Co., Ltd. The only information labelled on the packages were the participant ID and experiment date. The mean interval between the two study visits was 6.9 ± 0.3 days. To minimize a practice effect, an equal number of the participants were assigned to the group who consumed the MCT meal at the first visit and to the other who consumed it at the second visit.

The participants fasted from 10:00 p.m. on the night prior to the study visit. They arrived in the morning, and blood was drawn to determine plasma ketone body levels (acetoacetate and β-hydroxybutyrate) at baseline. They then consumed the test meal and rested for 90 min, after which blood was drawn again. Thereafter, the fMRI experiment was carried out. On one of the two visits, morphological MRI data were acquired for the VBM analysis. Additionally, the MMSE was assessed on the first visit before subjects consumed the test meal in order to measure baseline cognitive function level free from the effect of diet composition. Plasma ketone body levels were measured by an enzymatic method at SRL Inc. (Tokyo, Japan), who were blind to the subject information or time of blood sampling.

### 2.3. Cognitive Measures

The participants underwent fMRI scanning while performing two executive function tasks—namely, N-back and Go-Nogo tasks, which measure working memory and inhibitory control functions, respectively [25,27,38]. These tasks were chosen because they have been used to assess executive functions in the elderly, including those suffering from cognitive impairment [39,40,41,42,43], and importantly, these tasks can be performed with an MRI scanner [44,45].

**Figure 1:**
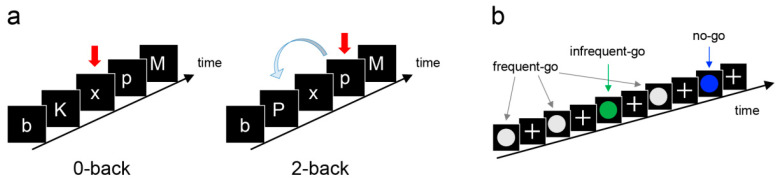
Schematic illustration of fMRI tasks. (**a**) N-back task. Participants should identify target letters (red arrow), which is the letter “x” (0-back condition) or the repeated letter that was shown 2 steps earlier (2-back condition). (**b**) The Go-Nogo task contained three types of trials: frequent-go, infrequent-go, and no-go trials. Participants should respond with button press in (frequent/infrequent) go trials and should not respond in no-go trials.

The N-back task was a block design letter N-back task [45,46]. This task contained a resting condition and two stimulus conditions: (1) in the “0-back” condition, the participants had to press a button each time they saw the letter X (Figure 1a); and (2) in the “2-back” condition, they had to press a button when the letter they saw was the same as the letter seen two letters before. This condition requires working memory. The letter stimuli were chosen from a set of 18 letters (all consonants except L, W, Y), ignoring upper- or lowercase. Each letter stimulus appeared for 500 ms, and the inter-stimulus interval was 1500 ms. Each stimulus block consisted of 20 stimuli containing seven targets and was indicated by cue displayed for 2 s before each block. Stimulus blocks and resting periods alternated within the experiment with a total of four 2-back and four 0-back blocks, with a defined sequence (0-2-0-0-2-2-0-2). During resting periods, the participants were instructed to fixate on a cross in the centre of the screen for 20 s.

The Go-Nogo task consisted of three types of trials: frequent-go, infrequent-go, and no-go trials [44,47]. In the frequent-go and infrequent-go trials, the participants were required to press a button, and in the no-go trial, the participants were required not to press the button, i.e., withhold the prepotent response tendency (inhibitory control). In each trial, a coloured circle was presented for 400 ms, and then a fixation cross was presented for 400 ms (Figure 1b). The time window to respond was 800 ms. The presentation frequency of infrequent-go trials was set equal to that of no-go trials so that brain responses to these trials were the same in regard to processing of infrequent stimuli but different in regard to inhibitory control load. To exclude potentially premature responses, go trials with response times of <150 ms were excluded from the analysis. The colour of the circle indicated the type of trial: grey indicated the frequent-go trial, whereas green and blue indicated the infrequent-go and no-go trials, respectively. The relationship between colour (green/blue) and trial type (infrequent-go/no-go) was counter-balanced across participants. One run consisted of 192 trials (142 frequent-go, 24 infrequent-go, and 24 no-go), and the filler frequent-go trials were also presented at the beginning and end of each run (15 trials at the beginning and 10 trials at the end). Each participant completed three runs.

### 2.4. Neuroimaging Measures

Functional imaging was conducted using a 3-Tesla MR system (Trio, Siemens, Erlangen, Germany), and gradient echo T2*-weighted echo-planner images (EPI) were acquired with BOLD contrasts. Forty-two contiguously interleaved transverse EPI-image slices covering the whole brain were acquired in each volume (slice thickness, 3 mm; no gap; repetition time, 3000 ms; echo time, 25 ms; flip angle, 83°; field of view, 192 mm^2^; matrix, 64 × 64). For each participant, data were acquired in four scanning runs (one for the N-back task and three for the Go-Nogo task). For the N-back task, 178 volumes (plus three “dummy” volumes) were acquired. Dummy volumes were included to allow for the T1 equilibrium effect. Similarly, 73 volumes (plus 2 dummy volumes) were acquired in each of the Go-Nogo task runs. Dummy volumes were discarded without analysis.

For the VBM analysis, three-dimensional (3D) T1-weighted images were scanned in the sagittal plane (slice thickness, 1 mm; repetition time, 1380 ms; echo time, 2.6 ms; flip angle, 15°; field of view, 260 mm^2^; matrix, 256 × 256), yielding 192 contiguous slices through the head.

In addition to functional and 3D T1-weighted images, T2-weighted images were also collected. Analysis using these images will be reported elsewhere.

### 2.5. Statistical Analysis

#### 2.5.1. Behavioural Data Analysis

To determine whether MCT meal ingestion effectively elevated plasma ketone body levels, ketone body levels pre- and post-intake of the test meals were compared using paired *t*-tests for each of the test meals. We evaluated the effect of the MCT meal on cognitive tasks using linear mixed model analyses. These analyses used scores on the cognitive tasks as the dependent variable and the meal (MCT/placebo) as an independent variable. In addition, other potential confounding factors, such as the day (1st/2nd visit), sex, and age, were included as independent variables to compensate for the effects of these factors. To adjust for the effect of repeated measures (two visits per participant), participant ID numbers were used as a random effect that affected the intercept. For the N-back task, the hit rate in the 2-back condition was used as the dependent variable. Similarly, for the Go-Nogo task, accuracy in the no-go trials was used as the dependent variable. We first analysed data from all participants together. Then, we stratified participants into high or low levels of global cognitive function groups based on their MMSE scores. The high group comprised participants who received full marks on the MMSE, while the low group comprised those who scored less (high, *n* = 11; low, *n* = 9). For each of these groups, linear mixed model analyses for N-back/Go-Nogo tasks were conducted using the models explained above. All statistical analyses of behavioural data were performed using R software v. 3. 6. 1 (www.r-project.org).

#### 2.5.2. fMRI Preprocessing and Analysis

fMRI data were analysed with SPM12 v7219 (http://www.fil.ion.ucl.ac.uk/spm/). The following preprocessing procedures were performed on data from the N-back and Go-Nogo tasks: correction for head motion, adjustment of acquisition timing across slices, spatial normalization using the anatomical image and the Montreal Neurological Institute (MNI) template, and smoothing using a Gaussian kernel with a full-width at half-maximum of 8 mm.

For the N-back task, analyses were performed in the context of the general linear model (GLM) using a two-level approach [48]. On the first level of analysis, condition blocks of 2-back and 0-back and the instruction cue were modelled as regressors of interest. To account for movement-related variance, the realignment parameters were also added as regressors of no interest in the design matrix. To extract BOLD signal changes induced by working memory load, contrast images for 2-back versus 0-back were computed and taken to the second level for random effects inference. On the second level, a paired t-test was used for comparison between experimental conditions (MCT vs. placebo meal). As noted earlier, because of its known relation to executive function, we focused our analysis on the DLPFC. Region of interest (ROI) analysis was performed using the Marsbar toolbox for SPM [49]. Practically, the bilateral middle frontal gyrus ROI was defined based on the automated anatomical labelling (AAL) atlas [50]. The above analysis steps were the same in both whole sample analysis and stratified analyses.

Analysis of the Go-Nogo task was performed using a similar two-level approach. On the first level of analysis, the event timing of the infrequent-go, the correct no-go, and the incorrect no-go trials were modelled as regressors of interest using the canonical haemodynamic response function provided by SPM, together with run-specific indicator and realignment parameters as regressors of no interest. The frequent-go trials were used as a baseline and were not coded as regressors [47]. To extract BOLD signal changes induced by inhibitory control, a contrast of the no-go versus infrequent-go trials was computed and taken to the second level for random effects inference. On the second level, a paired t-test was used for comparison between experimental conditions (MCT vs. placebo meal). Again, we focused our analysis on the DLPFC. This time, the ROI was set to the right DLPFC since a previous meta-analysis had shown that DLPFC involvement in inhibitory control is right-dominant [51]. Practically, the right middle frontal gyrus ROI was defined based on the AAL atlas. The above analysis steps were the same in both whole sample analysis and stratified analyses.

#### 2.5.3. VBM Preprocessing and Analysis

VBM analysis was conducted using data from all of the participants. Because each subgroup (high/low) did not have enough participants for this kind of analysis, we avoided the stratified analyses. The following preprocessing steps were performed using the CAT12 toolbox (CAT12.2, r1290) for SPM (http://www.neuro.uni-jena.de/cat/) with the default settings. Three-dimensional T1-weighted MRI images were normalized to MNI space, corrected for bias field homogeneities, and then segmented into grey matter, white matter, and cerebrospinal fluid. The amount of volume changes due to spatial registration was scaled to retain the original local volumes. The modulated images were smoothed using 8 mm full-width-half-maximum Gaussian smoothing using SPM12. We performed a multiple regression analysis using a GLM to identify brain regions whose grey matter volume was associated with the degree of improvement in cognitive task performance from the MCT meal. As reported below, such improvements were found in the N-back task. Hence, the degree of improvement was calculated by subtracting the hit rate in the 2-back condition under placebo meal from that under MCT meal and entered as a regressor. In addition, age, sex, and total intracranial volume were included in the GLM as regressors of no interest to control for the effects of these variables. For statistical inference, as we focused our analysis on the DLPFC, small volume correction was applied using the DLPFC ROI (*p* < 0.05 FWE-corrected voxel level). DLPFC ROIs were defined as a 5 mm sphere around the left middle frontal gyrus coordinate (-39, 51, -15) previously reported to be associated with working memory performance in a large-scale VBM study with over 1000 participants [38].

## 3. Results 

### 3.1. Ketone Body Levels

**Figure 2:**
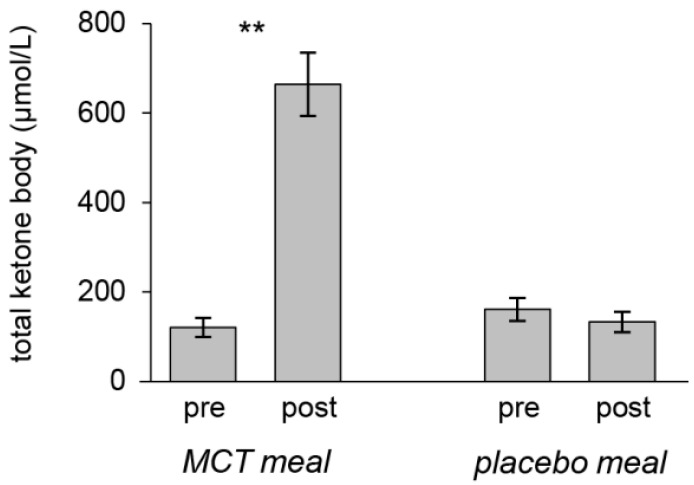
Mean plasma ketone bodies (acetoacetate plus *β*-hydroxy butyrate) at pre- and post-intake of the test meals. The MCT meal significantly elevated plasma ketone body levels. Error bars depict SEM; ** *p* < 0.01.

Figure 2 shows changes in plasma ketone body levels after consuming the MCT or placebo meal. As expected, the MCT but not the placebo meal significantly increased plasma ketone body levels.

### 3.2. Cognitive Measures and Neuroimaging Results from the Whole Sample Analysis

**Figure 3:**
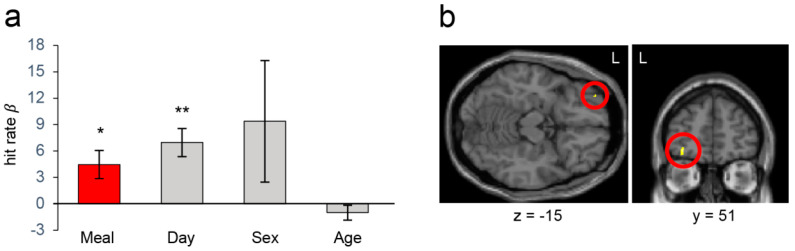
Behavioural and VBM results of analyses that included data from all participants. (**a**) The effects (β weights) on the hit rate in the 2-back condition in the N-back task based on meal (MCT/placebo), day (1st/2nd visit), sex, and age. Consuming the MCT meal significantly improved the hit rate. Error bars depict SEM; * *p* < 0.05, ** *p* < 0.01. (**b**) A region in the left DLPFC whose grey matter volume showed a significantly negative correlation with the degree of hit rate improvement by the MCT meal (red circle). L: left.

When the entire sample was analysed, working memory performance was significantly better after MCT meal intake than after placebo meal intake. The mean hit rates in the N-back task (2-back condition) after the MCT meal and after the placebo meal were 77.86 ± 2.99 and 73.39 ± 3.57% (mean ± SEM), respectively. Figure 3a shows the results of the linear mixed model analysis showing that consuming the MCT meal significantly increased the hit rate compared with placebo meal when the effect of confounding factors (i.e., day, sex, and age) were regressed out (*β* = 4.46, *p* < 0.05). Although fMRI analysis did not show corresponding BOLD signal changes with MCT meal consumption, the VBM analysis, which assessed the relationship between the degree of performance improvement and grey matter volume of the DLPFC, revealed that the participants who showed greater performance improvements had a smaller volume of the left DLPFC (*p* < 0.05, small volume corrected; Figure 3b). Performance improvements with the MCT meal were not significant in the Go-Nogo task (data not shown).

### 3.3. Cognitive Measures and Neuroimaging Results in the Stratified Analyses

**Figure 4:**
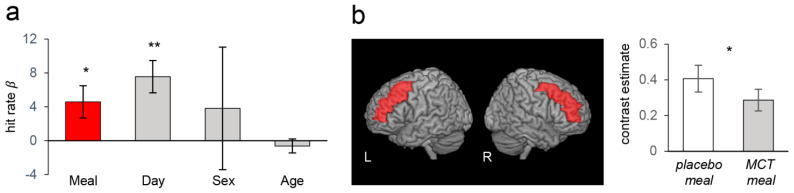
Behavioural and fMRI results of analyses in the high group. (**a**) The effects (β weights) on the hit rate in the 2-back condition in the N-back task based on meal (MCT/placebo), day (1st/2nd visit), sex, and age. Consuming the MCT meal significantly improved the hit rate. Error bars depict SEMs; * *p* < 0.05, ** *p* < 0.01. (**b**). fMRI BOLD analysis. Left: bilateral DLPFC region of interest (ROI) used in the BOLD analysis (red areas). Right: Consuming the MCT meal significantly decreased the BOLD response to the working memory load during the N-back task. * *p* < 0.05.

Analysis of the subgroups based on the global level of cognitive function (assessed by the MMSE) at baseline indicated that different aspects of executive function (working memory or inhibitory control) were improved by the MCT meal depending on the level of global cognitive function. The participants in the high group showed improved working memory performance. The hit rates of the N-back task with the MCT and placebo meals were 83.11 ± 3.34 and 79.22 ± 3.66% (mean ± SEM), respectively. Figure 4a shows the results of the linear mixed model analysis showing that consuming the MCT meal significantly improved the hit rate when the effects of confounding factors were regressed out (*β* = 4.58, *p* < 0.05). Corresponding to this performance improvement, as hypothesized, the fMRI analysis showed that the working memory load induced a significantly smaller BOLD signal increase in the bilateral DLPFC when the participants consumed the MCT meal (paired *t*-test, *p* < 0.05; Figure 4b).

**Figure 5:**
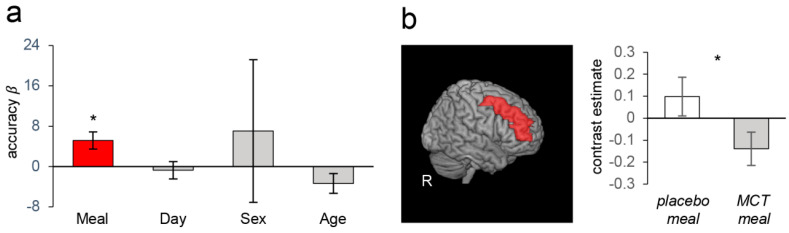
Behavioural and fMRI results of analyses in the low group. (**a**) The effects (β weights) on the accuracy in the no-go trials based on meal (MCT/placebo), day (1st/2nd visit), sex, and age. Consuming the MCT meal significantly improved the accuracy. Error bars depict SEMs; * *p* < 0.05. (**b**). fMRI BOLD analysis. Left: right DLPFC region of interest (ROI) used in the BOLD analysis (red area). Right: Consuming the MCT meal significantly decreased the BOLD response to the inhibitory control load during the Go-Nogo task. * *p* < 0.05.

In contrast, the participants in the low group showed significantly higher scores on the Go-Nogo task after the MCT meal than after the placebo meal (77.62 ± 5.59 vs. 72.53 ± 6.32%, respectively), indicating better inhibitory control performance. Figure 5a shows the results of the linear mixed model analysis showing that consuming the MCT meal significantly improved the score when the effects of confounding factors were adjusted for (*β* = 5.17, *p* < 0.05). Corresponding to this performance improvement, as hypothesized, the fMRI analysis showed that the inhibitory control load induced a smaller BOLD signal increase in the right DLPFC when the participants consumed an MCT meal (paired *t*-test, *p* < 0.05; Figure 5b). The participants in the high group showed no significant difference in Go-Nogo task performance (data not shown), and those in the low group showed no significant difference in N-back task performance (data not shown) that was dependent on the meal.

## 4. Discussion

In the present study, we examined the effect of MCT meal intake on cognitive functions in healthy elderly people and its underlying functional/structural brain mechanism. The results show that consuming the MCT meal was associated with better performance in some executive functions in our elderly subjects, depending on their baseline cognitive function. As expected, the brain region responsible for those cognitive functions (DLPFC) showed relatively decreased task-related BOLD signal changes when subjects consumed MCT meals, suggesting that the ketone bodies were consumed as an extra brain energy source. Furthermore, we found morphological characteristics of the brain associated with the effects of the MCT meal.

For the effect of MCT intake on plasma ketone body levels, we successfully observed significant elevation to a level comparable with that reported in previous studies [3,5,11]. Since we used high-fat/low-carbohydrate meals similar to what is used for a traditional ketogenic diet in the placebo condition, it was possible that this condition also induced a slight increase in ketone bodies. However, as the results show, this was not the case. Thus, we assume that any differences observed in cognitive performance/brain changes between the conditions are attributable to extra ketone bodies provided by the MCT meal. Previous studies have shown that the brain utilizes energy from ketone bodies as a function of its plasma level, and it is suggested that under the plasma level of 0.3–0.5 mM, the brain receives 3–5% of its total energy from ketone bodies [52]. Hence, as a similar degree of plasma ketone elevation was observed in the present study, the brain would have received at least 3–5% extra energy from ketone bodies. This would have contributed to the better performance in the cognitive tasks.

When the data from the entire sample were analysed, performance on working memory was significantly better after MCT meal intake than after placebo meal intake. This was prominent in the participants with small grey matter volume in the left DLPFC region. This region’s grey matter volume has been shown to positively correlate with working memory performance [38]. It is well known that VBM can be used to evaluate grey matter reduction due to the normal aging process and/or the progression of Alzheimer’s disease, and such reductions are supposed to reflect neurodegeneration or neural loss [27,53]. Because the participants in the present study were healthy elderly people, a small grey matter volume possibly reflected neural loss due to aging. Thus, we speculate that the participants with relatively reduced neurons benefited more from the extra energy source supplied by ketone bodies. Such beneficial effects may have arisen, at least in part, by improving mitochondrial energy metabolism [15,54]. Although we did find better cognitive performance associated with MCT meal consumption and its brain morphological correlates, we failed to find statistically significant changes in the accompanying BOLD signal in the analysis of the entire sample. As our stratified analyses indicated, however, the participants differed in which of the executive functions (i.e., working memory or inhibitory control) showed better performance associated with the MCT meal ingestion. Hence, analysing all participants together could have hindered the detection of significant changes in each of the individual executive functions.

Analysis of subgroups divided by baseline levels of cognitive function indicated that, in the participants with higher global cognitive function levels, consuming the MCT meal was associated with better performance in the working memory task, while participants with relatively lower global cognitive function levels showed such a relation in the inhibitory control task. At the same time, as hypothesized, the brain regions responsible for those executive functions (i.e., DLPFC) showed smaller task-induced fMRI BOLD signal increases. This indicates that neurons in this region utilized ketone bodies provided by MCTs as an extra energy source, and this underlies the better performance.

We hypothesize that when taking MCTs, the subsequent changes could have occurred in the DLPFC. First, as MCTs supplied abundant ketone bodies that could have been consumed as extra energy sources, the dependence of neural activity on glucose exhibited a relative decrease [55,56,57,58]. This lowered the degree of regional CBF increase [22,54], which in turn led to a reduced BOLD signal. Our findings are in line with those of previous studies showing that a heightened plasma ketone body level promotes ketone utilisation as an extra energy source in the brain [55,56,57,58], and we extended those findings by showing that such changes do occur in brain regions responsible for cognitive function at the time those functions are exerted. Thus, our findings might contribute to expanding our understanding of the physiological brain mechanisms underlying the association between MCT meal intake and changes in cognitive functions in elderly individuals.

The MCT meal was effective on different aspects of executive functions depending on the baseline level of cognitive function. The evidence related to this issue is still scarce because previous studies have mostly focused on the effects of MCT meals in patients with cognitive impairment, and data in healthy elderly individuals are scarce [5,9,10,11,12,59]. Some studies have indicated that the effects of MCT meals depend on the genotype [5,10]. Thus, it is possible that healthy elderly individuals also show individual differences along various dimensions. In this regard, our findings provide valuable insight into who would gain what kind of benefit from consuming MCT meals. Such insight is vital for the practical use of MCT meals and warrants further investigation.

There are several limitations in the study. The sample size was small, which could have resulted in false-negative results of the effect of the MCT meal in cognitive tests and neuroimaging indices. In addition, we subdivided the participants into two groups that included 11 higher and 9 lower global cognitive function participants, and the number of participants in the subgroups became even smaller. Nevertheless, we found statistically significant results in some cognitive tests and neuroimaging indices. This positive evidence from the current crossover study should be expanded by future studies by including a larger number of participants over a longer period of time to determine the possible causal effect of MCT meals on cognition by altering brain dynamics. In such large-scale studies, it is also important to measure and consider factors that might influence the effect of MCT meals, such as a participant’s history of gastrointestinal diseases and environmental/dietary factors, which were not covered in the present study. Another limitation was that in assessing brain dynamics, we employed tasks covering only two aspects of executive function (working memory and inhibitory control). Future work is needed to determine whether our findings can be extended to other aspects of executive function, such as cognitive flexibility. Additionally, in the present study, the working memory task (N-back) showed significant practice effects. By counterbalancing the order of MCT/placebo meal intake in the first and second visits, we effectively minimized the impact of this effect on the results. Nevertheless, it is advisable that future studies employ additional precaution, for example, placing a longer interval between visits. Finally, since we measured brain structural data once for each subject in the present study, it was impossible to directly assess whether consuming MCT meals (vs. placebo) induce structural changes in grey matter. This issue is an important avenue for future longitudinal studies.

## 5. Conclusions

In conclusion, in the present study, we found that oral intake of MCT was associated with heightened cognitive functions in healthy elderly individuals, and when these functions were exerted, the brain regions responsible for those functions showed BOLD signal changes indicative of the consumption of ketone bodies as an extra energy source. This is the first evidence showing brain activity changes at exactly the time when elderly subjects are engaged in cognitive activities that change with MCT intake. The results of this study indicate that there is a potential beneficial impact of ketone bodies with respect to improved cognitive outcomes due to an MCT meal, and further longitudinal studies with a larger cohort need to be conducted to confirm this outcome.

## Figures and Tables

**Table 1:** Demographics of participants.

	Mean ± SD
Age (years)	65.7 ± 3.9
Sex (M:F)	6:14
MMSE	29.75 ± 0.55
BMI	22.59 ± 2.56

Note. MMSE: Mini-Mental State Examination, BMI: body mass index.

**Table 2:** The components of Meiji817-B and placebo meals.

		Meiji817-B (50 g) *	Placebo (50 g)
Calorie (kcal)		371	371
Protein (g)		7.5	7.5
Carbohydrate (g)		4.4	4.4
Total lipids (g)		35.9	
	MCTs (g)	19.9	0
	LCTs (g)	16.0	35.9

MCTs: medium-chain triglycerides, LCTs: long-chain triglycerides. Meiji817-B includes 30.3 g caprylic acid and 9.8 g capric acid as medium-chain fatty acids per 100 g total fatty acids. * Representative values.

## Data Availability

The data presented in this study are available on request from the corresponding author when it is permitted by the ethics committee.
